# Micronutrient deficiencies in patients with chronic atrophic autoimmune gastritis: A review

**DOI:** 10.3748/wjg.v23.i4.563

**Published:** 2017-01-28

**Authors:** Federica Cavalcoli, Alessandra Zilli, Dario Conte, Sara Massironi

**Affiliations:** Federica Cavalcoli, Alessandra Zilli, Dario Conte, Sara Massironi, Gastroenterology and Endoscopy Unit, Fondazione IRCCS Ca’ Granda Ospedale Maggiore Policlinico, 20122 Milan, Italy; Federica Cavalcoli, Alessandra Zilli, Dario Conte, Postgraduate School of Gastroenterology, Department of Pathophysiology and Transplantation, Università degli Studi di Milano, 20122 Milan, Italy

**Keywords:** Chronic atrophic autoimmune gastritis, Nutritional deficiency, Vitamin B12, Iron, Vitamin C, Vitamin D, Calcium, Malabsorption

## Abstract

Chronic atrophic autoimmune gastritis (CAAG) is an organ-specific autoimmune disease characterized by an immune response, which is directed towards the parietal cells and intrinsic factor of the gastric body and fundus and leads to hypochlorhydria, hypergastrinemia and inadequate production of the intrinsic factor. As a result, the stomach’s secretion of essential substances, such as hydrochloric acid and intrinsic factor, is reduced, leading to digestive impairments. The most common is vitamin B12 deficiency, which results in a megaloblastic anemia and iron malabsorption, leading to iron deficiency anemia. However, in the last years the deficiency of several other vitamins and micronutrients, such as vitamin C, vitamin D, folic acid and calcium, has been increasingly described in patients with CAAG. In addition the occurrence of multiple vitamin deficiencies may lead to severe hematological, neurological and skeletal manifestations in CAAG patients and highlights the importance of an integrated evaluation of these patients. Nevertheless, the nutritional deficiencies in CAAG are largely understudied. We have investigated the frequency and associated features of nutritional deficiencies in CAAG in order to focus on any deficit that may be clinically significant, but relatively easy to correct. This descriptive review updates and summarizes the literature on different nutrient deficiencies in CAAG in order to optimize the treatment and the follow-up of patients affected with CAAG.

**Core tip:** Chronic atrophic autoimmune gastritis is an autoimmune disease characterized by progressive parietal cells destruction leading to hypochlorhydria and intrinsic factor deficiency. These alterations may result in vitamin B12 deficiency and iron malabsorption. A possible role of chronic atrophic autoimmune gastritis in the development of several nutritional deficiencies (*e.g*., calcium, vitamin D, vitamin C) has been reported. However, the prevalence and clinical impact of these deficiencies has not been elucidated. The present paper aims at investigating the relevance, frequency and clinical presentation of nutritional deficiencies in chronic atrophic gastritis to enable clinicians to promptly identify and correct any possible nutritional impairment.

## INTRODUCTION

Chronic atrophic autoimmune gastritis (CAAG) is an organ-specific autoimmune disease characterized by the presence of autoantibodies against gastric parietal cells and intrinsic factor[1,2]. CAAG occurs in approximately 2% of the general population, with a higher prevalence in elderly females[3]. Early alterations are characterized by the chronic inflammation in the submucosa that extends into the lamina propria of the mucosa, with a progressive destruction of both gastric and zymogene cells[4]. In the advanced stages of the disease, the gastric mucosa presents a dramatic reduction or absence of gastric glands. In particular, the parietal cells and zymogenic cells are absent from the gastric mucosa and are replaced by intestinal metaplasia[4,5]. These histological changes result in achlorhydria, hypergastrinemia and intrinsic factor deficiency[6].

The reduction of intrinsic factor levels results in vitamin B12 malabsorption. Indeed, intrinsic factor has a key-role in binding vitamin B12 in the duodenum and transporting it to the terminal ileum for absorption[7,8]. More recently, iron deficiency and iron deficiency anemia have been reported in the setting of CAAG, particularly in younger patients[9].

Moreover, the deficiency of several vitamins and micronutrients, such as vitamin C, vitamin D, folic acid and calcium, has been described in patients with CAAG[10,11] or long-standing achlorhydria due to proton pump inhibitors (PPIs) therapy or gastrectomy[12]. The pathogenic mechanism underlying these changes seems to be the increased destruction or decreased absorption of nutrients in the gastric mucosa, possibly due to the elevated pH and bacterial overgrowth[11].

This paper aims to critically review the current knowledge on CAAG and nutritional deficiency in order to focus on the existence of any deficit that may be clinically significant, and to optimize the treatment and the follow-up of patients affected with CAAG.

## MICRONUTRIENT DEFICIENCIES

### Vitamin B12

Pernicious anemia (PA) is the most common cause of megaloblastic anemia in Western countries, resulting from impaired cobalamin (*i.e*., vitamin B12) absorption due to the lack of intrinsic factor production caused by the destruction of parietal cells. Cobalamin cannot be synthesized in the human body, but should be introduced with food, where it is bound to proteins. After hydrolysis by gastric pepsin and chlorhidric acid, cobalamin binds to the intrinsic factor, which is released by the gastric parietal cells and is essential for its absorption in the distal ileum[13,14]. As a cofactor for two enzymes, the adenosylcobalamin-dependent methylmalonyl-CoA mutase in mitochondria and the methylcobalamin-dependent methionine synthase in the cytoplasm, cobalamin is important in several biological processes, such as DNA synthesis and regulation, energy production and erythropoiesis[15]. The spectrum of diseases associated with vitamin B12 deficiency is very wide, varying from asymptomatic to life-threatening pancytopenia or myelopathy[16]. Vitamin B12 deficiency can cause macrocytic anemia, mild hyperhomocysteinemia that however increase the risk of atherothrombosis, neuropsychiatric manifestations, as paraesthesia, weakness, gait abnormalities, and cognitive or behavioral changes (Table 1).

**Table 1:** Clinical and laboratory findings in vitamin B12 deficiency

General symptoms	Weight loss observed in most patients
	Low-grade fever occurs in one third of newly diagnosed patients and promptly disappears with treatment
Gastrointestinal symptoms	Smooth tongue (50% of patients) with loss of papillae. Changes in taste and loss of appetite
	Patients may report either constipation or having several semi-solid bowel movements daily
	Anorexia, nausea, vomiting, heartburn, pyrosis, flatulence and a sense of fullness
Brain	Altered mental status. Cognitive defects (“megaloblastic madness”): depression, mania, irritability, paranoia, delusions, lability
Sensory organs	Optic atrophy, anosmia, loss of taste, glossitis
Bone marrow	Hypercellular bone marrow
	Increased erythroid precursors
	Open, immature nuclear chromatin
	Dyssynchrony between maturation of cytoplasm and nuclei
	Giant bands, metamyelocytes
	Karyorrhexis, dysplasia
	Abnormal results on flow cytometry and cytogenetic analysis
Spinal cord	Myelopathy
	Spongy degeneration
	Paresthesias
	Loss of proprioception: vibration, position, ataxic gait, limb weakness/spasticity (hyperreflexia)
	Positive Romberg sign
	Lhermitte’s sign
	Segmental cutaneous sensory level
Autonomic nervous system	Postural hypotension
	Incontinence
	Impotence
Peripheral nervous system	Cutaneous sensory loss
	Hyporeflexia symmetric weakness
	Paresthesias
Genitourinary symptoms	Urinary retention and impaired micturition may occur because of spinal cord damage. This can predispose patients to urinary tract infections
Reproductive system	Infertility
Abnormalities in infants and children	Developmental delay or regression, permanent disability
	The patient does not smile
	Feeding difficulties
	Hypotonia, lethargy, coma
	Hyperirritability, convulsions, tremors, myoclonus
	Microcephaly
	Choreoathetoid movements, peripheral blood
	Macrocytic red cells, macro-ovalocytes
	Anisocytosis, fragmented forms
	Hypersegmented neutrophils
	Leukopenia, possible immature white cells
	Thrombocytopenia
	Pancytopenia
	Elevated lactate dehydrogenase level (extremes possible)
	Elevated indirect bilirubin and aspartate aminotransferase levels
	Decreased haptoglobin level
	Elevated levels of methylmalonic acid, homocysteine, or both

Recent epidemiological studies support the evidence that CAAG and PA are found across all the continents[17,18] and are probably under-diagnosed, since most patients with microcytic or macrocytic anemia are treated with iron, folates, and cobalamin, without any investigation of other causes of anemia. A study, which analyzed blood B12 levels in 729 American people aged 60 years or older, observed that 1.9% of the survey population had unrecognized and untreated PA: the prevalence was 2.7% in women, 1.4% in men and similar in black and white women (4.3% *vs* 4.0%)[19]. In the literature the prevalence of vitamin B12 deficiency among elderly people can range between 5% and 40% depending on the cut-off value of vitamin B12 used: the most frequent serum cut-off to diagnose vitamin B12 deficiency is 150 pmol/L (corresponding to 203 pg/mL)[20-22]. Autoimmune gastritis (pernicious anemia) is the most common cause of severe vitamin B12 deficiency due to food-cobalamin malabsorption in the elderly, nevertheless use of medications, as proton pump inhibitors, histamine H_2_ blockers, metformin or cholestyramine can interfere with or reduce vitamin B12 absorption. Although autoimmune gastritis is known to be a major cause of vitamin B12 deficiency, the exact prevalence of vitamin B12 deficiency in CAAG has not been fully elucidated, being reported in a percentage varying from 37% to 69% of the cases (Table 2)[23-27], this probably being due to the high heterogeneity of the populations considered and the limited availability of prospective studies. Moreover, chronic *Helicobacter pylori* (*H. pylori*) infection is frequently associated with atrophic gastritis and a study reported that *H. pylori* was found in 56% of people with vitamin B12 deficiency[28]. Pernicious anemia accounts for 15% to 25% of vitamin B12 deficiency in elderly people[29]. In a study on 296 Chinese patients, PA was diagnosed in 61% of the patients having megaloblastic anemia with vitamin B12 or folate deficiency[30].

**Table 2:** Demographic and biochemical characteristics of chronic atrophic autoimmune gastritis patients with vitamin B12 deficiency

**Ref**.	**Total No. of patients**	**Gender (M/F)**	**Age (yr), median**	**Gastrin (pg/mL), median**	**Prevalence Vit. B12 deficiency, *n* (%)**	**Vitamin B12 (pg/mL) median^1^**	**Prevalence of neurological complications**
Marignani et al[23], 1999	80	24/56	56	491	44 (55.0)	87.5	NA
Hershko et al[24], 2006	160	53/107	50	846	111 (69.4)	82.0	17%
Annibale et al[25], 2005	140	49/91	55	500	65 (46.5)	80.0	NA
Miceli et al[27], 2012	99	72/27	59	726	37 (37.4)	NA	6%
Lahner et al[26], 2015	83	42/41	59	NA	43 (51.8)	138.0	NA

1 Median vitamin B12 levels in patients with macrocytic anemia at presentation. NA: Not assessed.

The variability of vitamin B12 levels in CAAG seems to be influenced by a large number of genetic and environmental factors[31]. The genotypes HLA-DRB1*03 and DRB1*04, which are known to be associated with other autoimmune diseases (*e.g*., diabetes mellitus type 1 and autoimmune thyroid disease), were significantly associated with PA[32].

Recently, several studies about genome-wide association have shown that some single nucleotide polymorphisms (SNPs) are linked with the vitamin B12 serum/plasma levels. A meta-analysis of three genome-wide association scans on Caucasians has found genome-wide associations between plasma vitamin B12 and SNPs on the methylmalonyl-CoA mutase (MUT), the intrinsic factor-cobalamin receptor cubilin (CUBN), the transcobalamin I (TCN1) and the fucosyltransferase 2 (FUT2) genes[33]. A more recent study has showed that, among 14 SNPs associated with vitamin B12 levels, a genetic variant of transcobalamin II (TCN2), encoding for the transport proteintranscobalamin 2, and a genetic variant of fucosyltransferase 6 (FUT6), encoding for the enzymefucosyltransferase 6, were significantly more frequent in CAAG patients with PA compared to healthy controls[26]. This observation was in contrast with the association of this variant of FUT6 and higher vitamin B12 plasma levels detected in a genome-wide association study performed on the Chinese male population[34]. Furthermore, another study observed a link between *FUT* gene variants, especially *FUT2*, *FUT3* and *FUT6*, *H. pylori* status and intestinal-type gastric cancer risk[35,36].

Even if in healthy older adults the recommended therapy is the oral replacement of crystalline vitamin B12, the patients with CAAG and secondary vitamin B12 malabsorption, will require its parenteral replacement with intramuscular cyanocobalamin at a dose of 1000 μg daily for one week, then weekly for 4 to 8 wk, and then monthly for life. In case of mild deficiency with mild atrophic gastritis high-dose oral cyanocobalamin at 500 to 1000 μg daily can be considered adequate[16]. Concomitant iron and folate replacement is needed to achieve a full hemoglobin response[16].

Moreover, PA is frequently associated (up to 27% of cases) with iron deficiency anemia (IDA)[37]. In a study by Hershko et al[24] low serum vitamin B12 levels were found in 100% macrocytic, 92% normocytic and 46% microcytic patients with CAAG, whereas iron deficiency was found in all the patients with microcytic anemia, but also in 50% of the normocytic and 10% of the macrocytic patients. Thus, a considerable proportion of patients had combined iron and cobalamin deficiencies. The mean age was 41 ± 15 years in those CAAG patients presenting with IDA and 59 ± 16 years in those patients presenting with PA. Whilst autoimmune atrophic gastritis impairs both food iron and cobalamin absorption, age, sex, the co-presence of *H. pylori* infection, duration and severity of disease may determine the clinical presentation of CAAG as microcytic IDA or macrocytic megaloblastic anemia. In fact, in female patients presenting with IDA menstrual blood loss may have been an important role in the development of iron deficiency, aggravated by the inability to compensate it by improving food iron absorption. Co-existing *H. pylori* gastritis, which is more frequent in young patients, may contribute to the development of IDA. Conversely, the depletion of cobalamin stores may take many years longer and manifest in elderly patients, implying a severe impairment of the intrinsic factor secretion.

Finally, vitamin B12 is important to bone development, in particular for the osteoblastic function, as demonstrated by *in-vitro* studies[20,38] and population studies showing lower bone mineral density and greater fracture risk in patients with vitamin B12 deficiency[39,40]. In a two-year-long randomized controlled trial a 80% reduction in the hip fracture risk was observed among stroke patients after vitamin B12 repletion[41], probably due to hypergastrinemia, which has been shown to stimulate parathyroid activity in animal models and in humans, with the consequent hyperparathyroidism and increased bone turnover[42-44]. However, a study by Merriman et al[45] did not confirm these data, as no reduction of risk of hip fracture showed among patients with PA after B12 repletion, thus suggesting that the presence of mechanisms other than B12 deficiency mediated the fracture risk.

Therefore, PA does not only influence cobalamin plasma levels, but also iron absorption and bone development: therefore patients with CAAG should undergo the evaluation of martial pool, B12 stores and 25-OH vitamin D levels.

### Iron

Alimentary iron is available in two forms: heme and non-heme iron. Heme iron, present in meat hemoglobin and myoglobin, is in a ferrous form which is soluble at alkaline pH and is easily absorbed in the duodenum without any need for chelation. However, non-heme ferric form represents about 80% of dietary iron and is less absorbable. Ferric iron is insoluble, precipitates at pH > 3, and is not absorbed unless reduced to the ferrous or chelated form. Different observations have demonstrated the importance of gastric secretion of both hydrochloric and ascorbic acid in the solubilization and reduction of non-heme food iron for a normal iron absorption[24,46,47].

Moreover, a possible role of achlorhydria in the development of iron malabsorption has been suggested in different hypo/achlorhydria models[48].

A possible association between CAAG and iron malabsorption was initially proposed in 1930[7,49], and it was most commonly assumed that iron deficiency anemia was caused by achlorhydria-induced malabsorption of dietary iron. In 1963 Cook and colleagues observed a significant impairment of iron absorption in patients with PA, which was corrected by adding gastric juice[50]. On the other hand, later studies did not confirm those observations and reported that the achlorhydria-induced malabsorption of dietary iron was unlikely to be the primary etiology of iron deficiency[51,52]. In recent years, however, the co-existence of CAAG and iron deficiency anemia (IDA) has been increasingly reported. In 1966 Dagg[53] introduced the concept of IDA progressing to classic PA in CAAG patients, demonstrating a 19% prevalence of achlorhydria and anti-parietal cell antibodies (APCA) positivity in patients with IDA and predicting that 32% of such patients would develop PA. More recently[23], based on the observation of high prevalence of *H. pylori* positivity in young CAAG patients, it has been proposed that *H. pylori* infection may represent an early stage of gastritis. In later stages the infectious process is gradually replaced by an autoimmune disease with the irreversible destruction of the gastric body mucosa. Indeed, with the advancing age of presentation there is a progressive increase in the mean corpuscular volume of erythrocytes and in the severity of hypergastrinemia and cobalamin deficiency in CAAG patients[23]. Histological active chronic inflammation has been reported to be 4 times more common in patients with IDA than in classic PA patients presenting with macrocytosis, probably due to their higher rate of active *H. pylori* infection[23].

In 2002 Dickey[46] observed a significant proportion of IDA patients having a concomitant CAAG (8/41). None of such patients had concomitant vitamin B12 deficiency, suggesting that CAAG may present with IDA from the onset of the condition. In addition, 20%-40% of PA patients developed IDA after treatment with parenteral vitamin B12, those observations suggesting the presence of subclinical iron deficiency in patients with clinically manifest PA[46].

Moreover, a prospective study by Hershko et al[24] showed a high (27%) proportion of CAAG among patients with iron deficiency anemia (IDA) without any apparent gastrointestinal diseases. Differently from classic PA, most of these patients were women, 20 years younger and had coexistent *H. pylori* infection. The authors then postulated that iron deficiency would develop earlier than vitamin B12 deficiency in young women with menstrual blood loss[24]. Annibale et al[54-56] found atrophic body gastritis in the 27% of patients with refractory IDA without gastrointestinal symptoms, in accordance with previous studies[23]. The same authors in 1999 compared CAAG patients presenting with IDA with others presenting with PA, and found that in 45% of CAAG patients IDA was the first clinical manifestation of the disease[23]. Finally, some recent studies have reported the occurrence of IDA in pediatric patients affected by CAAG, highlighting that CAAG should be considered when investigating refractory iron deficiency anemia in children[9].

In conclusion, iron malabsorption and the onset of iron deficiency anemia appear to be biologically plausible and supported by different models of achlorhydria. Therefore, the patients with PA should be evaluated for concomitant iron deficiency development[57]. On the other hand, CAAG should be taken in consideration when evaluating patients with unexplained IDA, after the proper exclusion of any bleeding lesions.

Since patients with iron deficient anemia and autoimmune atrophic gastritis may be refractory to oral iron treatment, *H. pylori* eradication in combination with continued oral iron therapy have been suggested[23,24,54-56].

### Vitamin C

Ascorbic acid, which is important in the production of key proteins such as collagen, norepinephrine and serotonin, is not synthesized *ex-novo* by the human body, but can only be introduced through the diet and then absorbed in the stomach and along the entire length of the small intestine[58].

Ludden et al[11] noted that CAAG patients had a diminished absorption of ascorbic acid and suggested that was due to the destruction of ascorbic acid in the gastric mucosa because of elevated pH and bacterial overgrowth, as suggested by more recent studies showing destruction of ascorbic acid caused by hypochlorhydria induced by potent acid suppression[12,59].

Alt et al[60] evaluated the effect of pH on ascorbic acid stability *in vitro* and demonstrated the destruction of 65% of the ascorbic acid at pH 7.95 *vs* only 14% at pH 1.45.

Ascorbic acid is an important antioxidant that inhibits the generation of N-nitroso compounds (NOC) and scavenges nitrites in the gastric juice by converting them to nitric oxide[61,62]. The ability of ascorbic acid to scavenge nitrite depends on the ratio of vitamin C/nitrite and gastric pH, thus increased NOC levels are generated in case of a decreased ratio of vitamin C/nitrite and pH > 2-4[63]. Finally, since some population-based epidemiologic studies have showed negative correlations between the vitamin C intake and gastric cancer, ascorbic acid is thought to decrease the oxidative damage to the gastric mucosa by scavenging free radicals and NOCs and attenuating the *H. pylori*-related inflammation[10]. This antioxidant role may also reduce the inflammation present in the gastric mucosa of CAAG patients, although further studies are necessary to confirm this hypothesis.

### Calcium

Calcium is absorbed as calcium ion (Ca^2+^) in the proximal small intestine in both active and passive way. The absorption process begins in the stomach with the dissolution of calcium salts (*e.g*., calcium carbonate) to calcium chloride (CaCl_2_) which easily dissociates to Ca^2+^ which are highly water-soluble[64]. The bio-availability of dietary calcium salts depends on several factors, including the gastric acid secretion, physiological function of the stomach and intestine, levels of vitamin D in the tissues and circulation and the chemical structure and quantity of the calcium compounds ingested[65-67].

Gastric acid plays an important role as it increases the dissolution and ionization of poorly soluble calcium. It has been reported that conditions causing a decrease in gastric acid secretion, such as gastric surgery, use of PPIs and CAAG, lead to a reduction in the dissolution of calcium salts, which may not be properly absorbed[66].

There have been a few reports since the 1960s emphasizing the risk of malabsorption of calcium in patients with achlorhydria and atrophic stomach mucosa[66,68]. In 1985 Recker[66] compared the absorption of calcium carbonate and calcium citrate, measured by a modified double-isotope procedure, in normal subjects and patients with achlorhydria. The study showed that the absorption of calcium carbonate in patients with achlorhydria was significantly lower than in the normal subjects, supporting the role of gastric acid in calcium homeostasis. Calcium malabsorption has been demonstrated in animal models with gastric acid suppression[69,70], as well as in humans with achlorhydria under fasting conditions. However, the degree of calcium malabsorption in patients with CAAG remains controversial, as Eastell et al[71] have found normal calcium absorption with meals in patients with pernicious anemia and achlorhydria.

Interestingly, a few studies have reported the possible association between pernicious anemia and/or CAAG, and osteopenia and osteoporosis[71,72]. The study by Eastell et al[71] has reported a significant lower bone mineral density in post-menopausal women affected by pernicious anemia as compared to healthy controls. In the same study the authors showed that the decrease in bone mineral density was linearly related to a decline in serum levels of pepsinogen I, which is a serum biomarker of the structure and function of the gastric oxyntic mucosa. Its levels tend to decrease with the increase in the grade of atrophy of the oxyntic mucosa[71]. Another study, instead, has recently observed the reduction in bone mineral density and an increased frequency of osteopenia and osteoporosis in male, but not female patients with CAAG[73]. Moreover, the incidence of fractures has been reported increased in patients with pernicious anemia/CAAG[45,72]. On the other hand, Kakehasi et al[74] in a cross-sectional study did not find any significant difference in bone mineral densities between patients with autoimmune gastritis and *H. pylori* gastritis and the controls. However, it should be noted that the lack of significance in the reduction of bone mineral density might be due to the low number of patients with CAAG included in the study. In addition, a paper from the Finnish group has suggested that the administration of supplementary calcium in patients with osteoporosis may require the evaluation of gastric acid secretion, atrophic gastritis and the use of PPIs[64].

Further evidence on a possible role of gastric acidity in calcium absorption results from gastric surgery and the use of anti-acid drugs: in these setting an increased risk for low bone mass or fractures has been reported.^75^ Gastrectomy increases the risk of osteoporosis and fractures due to weight loss and changes of body composition as well as calcium malabsorption[75,76]. More recently, Krause et al[77] demonstrated a statistically significant increase of osteomalacia, marrow fibrosis, and impaired calcium distribution within the mineralized matrix in gastrectomized patients as compared to controls. Long-term acid-suppressive therapy can also raise the risk of fractures[78-82]. A meta-analysis revealed that proton pump inhibitors can increase the risk of hip, spine, and any site fractures by 30%, 56% and 16%, respectively[83].

In conclusion, even if biologically plausible, the evidence regarding the role of CAAG and/or hypochlorhydria in calcium malabsorption remains controversial; however, the increasing evidence on a relationship between CAAG/hypochlorhydria and the risk of osteoporosis suggests that in this setting an important impairment of bone mineralization exists and can be secondary to long-term calcium malabsorption.

### Vitamin D

To date a few studies have investigated the association between CAAG and vitamin D deficiency[84,85].

In 1992 Eastell et al[71] did not observe the presence of vitamin D deficiency in a group of 21 patients with CAAG as compared to healthy subjects. However, the very low number of cases considered have possibly limited the results of this study[71]. In 2012 a study[85] observed, for the first time, significantly lower 25-OH vitamin D levels in patients with CAAG as compared to non-specific gastritis or the general population. In this study, the 25-OH vitamin D mean concentration in subjects with CAAG was 9.8 ± 5.6 ng/mL (CI: 8.4-11.2) *vs* 21.3 ± 12.2 ng/mL (CI: 19.7-22.9) in healthy control subjects. Based on these observations, the authors hypothesized that hypovitaminosis D might be a risk factor for the development of autoimmune diseases[85]. More recently, a paper from our group[84] has reported the increased prevalence of hyperpathyroidism secondary to vitamin D deficiency in patients affected by CAAG. This finding can suggest that the regulation of calcium and/or vitamin D metabolism may be impaired in patients with CAAG, potentially because of the malabsorption of vitamin D in the intestine.

Moreover, as stated in the earlier Vitamin B12 and Calcium sections, different studies have reported on the risk of calcium malabsorbtion[64,66,68] and an increased risk of osteoporosis and pathological fractures in CAAG patients[45,72,86]. Remarkably, the active transcellular absorption of Ca^2+^ in the duodenum and proximal small intestine depends on vitamin D and represents the most important physiological pathway for the absorption of the calcium. Thus, it seems possible that vitamin D deficiency in CAAG patients can also explain calcium malabsorption and alterations in bone mineralization.

Further prospective studies are needed to fully elucidate the association between CAAG and vitamin D deficiency.

## CONCLUSION

Although pernicious anemia is the most frequent deficiency observed in patients with CAAG, the deficiency of other vitamins and micronutrients, such as vitamin C, vitamin D and calcium, has been described in the current literature (Table 3)[11,60]. The pathogenic mechanisms seem to be the increased destruction or decreased absorption of nutrients in the gastric mucosa, because of elevated pH or bacterial overgrowth[11].

**Table 3:** Summary of the main types of deficit described in chronic atrophic autoimmune gastritis patients

**Deficit**	**Mechanism of action**	**Effects**	**Reported prevalence**
Vitamin B12	Lack of intrinsic factor reduced vitamin B12 absorption in terminal ileum	Pernicious anemia	37%-69%[24,27]
		Neurological alteration	
		Osteopenia/osteoporosis	
Iron deficiency	Gastric acid increases the dissolution and ionization of poorly soluble calcium salt	Microcytic anemia	41%[24]
Vitamin C	Destruction of ascorbic acid in the gastric mucosa for elevated pH and bacterial overgrowth	Reduced and oxidative effects	Not known
Calcium	Gastric acid increases the dissolution and ionization of poorly soluble calcium salt	Osteopenia/osteoporosis	Not known
Vitamin D	Not clarified	Secondary hyperparathyroidism	12.1%[84]
		Osteopenia/osteoporosis	
		Increased incidence of autoimmune diseases	

Vitamin B12 deficiency, causing hematological and neurological consequences remains the most extensively investigated nutritional impairment in CAAG. However, even in this setting further studies appear to be necessary to clarify the exact prevalence of vitamin B12 deficiency in CAAG patients. Moreover, a large number of environmental and genetic factors (such as some single nucleotide polymorphisms on particular genes) may influence the levels of vitamins in patients with CAAG[31,26], and their impact remains to be fully elucidated. With regard to iron absorption, the current literature reports IDA occurrence in up to 27% of patients with PA, this suggesting that CAAG should be taken into consideration in patients with unexplained IDA, after the proper exclusion of potential bleeding lesions. On the other hand, it appears relevant to evaluate the iron status in CAAG patients at diagnosis and during follow-up[57].

Finally, as some reports suggest, CAAG may lead to an increased risk of osteoporosis and pathological fractures secondary to the deficiency of vitamin B12 and/or calcium and vitamin D: the prompt recognition of such deficiencies is crucial to reduce the risk of bone fractures.

Overall, the increasing amount of evidence on the possible occurrence of multiple vitamin deficiencies in CAAG, leading to hematological, neurological and skeletal manifestations, highlights the importance of an integrated evaluation of these patients.
